# Evaluation of the PreTect HPV-Proofer E6/E7 mRNA Assay for the Detection of Precancerous Cervical Lesions in the Greek Female Population

**DOI:** 10.3390/pathogens14090853

**Published:** 2025-08-28

**Authors:** Athanasia Kafasi, Vassiliki C. Pitiriga, Nikolaos Spanakis, Nikolaos Vlachos, Nikolaos Thomakos, Stamatios Stournaras, Athanasios Tsakris, Georgios Kaparos

**Affiliations:** 1 Department of Microbiology, Medical School, National and Kapodistrian University of Athens, 11527 Athens, Greece; 2 2nd Department of Obstetrics and Gynecology, Areteion Hospital, National and Kapodistrian University of Athens, 11528 Athens, Greece; 3 Division of Gynecologic Oncology, 1st Department of Obstetrics and Gynecology, Alexandra Hospital, National and Kapodistrian University of Athens, 11528 Athens, Greece; 4 Maternity and Gynecology Clinic, IASO Hospital, 15123 Athens, Greece; 5 Department of Microbiology, Areteion Hospital, National and Kapodistrian University of Athens, 11528 Athens, Greece

**Keywords:** cervical cancer screening, HPV mRNA testing, E6/E7 oncogenes, PreTect HPV-proofer

## Abstract

Cervical cancer remains a significant public health concern, ranking as the 10th most common cancer among women in Greece. Current screening programs primarily rely on cytology and HPV DNA testing; however, their positive predictive value (PPV) for detecting high-grade cervical intraepithelial neoplasia (CIN2+) remains limited. This study aimed to compare the clinical performance of the HPV mRNA test with that of the HPV DNA test, focusing on their PPV for detecting CIN1+ lesions in a cohort of Greek women. A total of 114 women undergoing routine cervical cancer screening were tested using an HPV DNA assay (detecting 41 HPV types), Pap smear, and were referred for colposcopy and biopsy when indicated. Among them, 29 women aged 18 to 65 years (mean age: 35.1 ± 10.8 years) who tested positive for one or more of the five high-risk HPV types (16, 18, 31, 33, 45) were further assessed using the PreTect HPV-Proofer^®^ mRNA assay. Of these 29 women, 11 (37.9%) had negative biopsy findings, 16 (55.2%) were diagnosed with CIN1, and 2 (6.9%) with CIN2, corresponding to a positive predictive value (PPV) of 55.2% for CIN1 and 6.9% for CIN2 with the HPV DNA test. Among the 17 women who tested positive for HPV mRNA, 13 were diagnosed with CIN1 and 2 with CIN2. Among the 12 women who tested negative for HPV mRNA, 3 had CIN1 and 9 had negative biopsy results. Based on these findings, the PPV of the HPV mRNA test for CIN1 was 76.5%, the negative predictive value (NPV) was 75.0%, and the clinical sensitivity for CIN1 was 81.3%. For CIN2, the PPV was 11.8%, while the clinical sensitivity and NPV were 100%. These findings highlight the potential of HPV mRNA testing to improve specificity in cervical cancer screening by more accurately identifying clinically significant lesions and reducing unnecessary colposcopies.

## 1. Introduction

Cervical cancer remains a major global health concern, ranking as the fourth most common cancer among women worldwide, according to the Catalan Institute of Oncology and the International Agency for Research on Cancer (ICO/IARC) Information Centre on HPV and Cancer (2023). In Greece, it ranks as the 10th most frequent cancer among women overall, and as the 3rd most frequent cancer among women aged 15 to 44 years [1].

Although cytology-based screening programs using the Papanicolaou test (Pap test) or Liquid-Based Cytology (LBC) have proven to be effective [2,3], in recent years, several countries have already replaced or are in the process of replacing these programs with HPV DNA testing programs to increase clinical sensitivity in detecting high-grade lesions [4,5,6,7]. However, the positive predictive value (PPV) of high-risk (HR) HPV DNA testing remains relatively low, as only a small proportion of women with a positive result actually have CIN2+ lesions at the time of testing [8].

To address this limitation, research has increasingly focused on alternative or complementary methods for risk stratification in HPV-positive women. Since the progression to cervical malignancy requires overexpression of the viral oncoproteins E6 and E7 [9], the detection of mRNA from these oncogenes may offer greater clinical specificity than HPV DNA testing in identifying CIN2+ lesions [10]. These mRNA transcripts are more stable and exhibit fewer nucleotide variations during viral DNA integration, which is often associated with high-grade lesions and cervical cancer. The use of LBC enhances HPV mRNA testing by providing a collection medium that adequately preserves RNA, thereby enabling in vitro amplification and detection, and supporting improved analysis and diagnostic accuracy [8].

Several studies have shown that the increased clinical specificity of the mRNA test makes it a valuable triage tool, capable of significantly reducing unnecessary colposcopies and biopsies [11,12,13,14,15]. For example, the study by Benevolo et al. [11], which compared the PreTect Proofer E6/E7 mRNA assay with HPV DNA testing in 1201 women, found that the clinical specificity of the mRNA test was 82% versus 29% for DNA testing in ASC-US cases, and 76% versus 13% in LSIL cases. mRNA triage reduced colposcopy referrals by 79% in ASC-US and 69% in LSIL cases, compared to only 38% and 15%, respectively, with DNA testing. Similarly, the study by Sørbye et al. revealed that among 311 women with ASC-US/LSIL cytology, HPV DNA positivity was 32.7%, whereas only 13.2% were mRNA-positive [12]. Women in the DNA-positive group had 2.8 times higher odds of being referred for colposcopy than those in the mRNA-positive group. In the study by Westre et al. [13], among 564 women undergoing ASC-US/LSIL triage, the 5-type mRNA test led to significantly fewer biopsy referrals (16%) compared to HPV DNA testing (25%).

In this study, we compared the performance of the HPV mRNA test with that of the HPV DNA test in terms of positive clinical predictive value, focusing on their ability to detect CIN1+ lesions in a cohort of Greek women. The objective was to assess the potential added value of the HPV mRNA test in enhancing the effectiveness of cervical cancer screening practices.

## 2. Methods

### 2.1. Study Design

In our study, 114 women aged 18 to 65 years were enrolled. All women were permanent residents of Greece and attended Areteion Hospital, Alexandra Hospital, and IASO Hospital for routine gynecological screening between May 2019 and February 2021.

Exclusion criteria included the following: age below 18 or above 65 years, pregnancy, history of hysterectomy, invasive treatment for CIN2+ within the past seven years, prior vaccination with the bivalent or nine-valent HPV vaccine before HPV DNA testing, a high-grade squamous intraepithelial lesion (HSIL) on the most recent Pap test, HIV infection, immunosuppression, chemotherapy within the last five years, and a history of genital warts infection.

Before the examination, each woman completed a demographic questionnaire, which included questions on age, place of residence in Greece, smoking habits, age at sexual initiation, number of children and age at childbirth, frequency of Pap testing, and HPV vaccination status. Data on educational level, economic status, number of sexual partners, and use of protection during sexual activity were not collected.

All women underwent cervical cancer screening via co-testing (Pap test and HPV DNA test), in line with the 2019 ASCCP guidelines for cases in which the HPV DNA test used is not FDA-approved for primary screening. Cervical samples were collected using either the digene^®^ Hybrid Capture 2 (HC2) DNA Collection Device (QIAGEN, Venlo, The Netherlands)—which includes a cervical brush and transport medium—or a cervical brush with CellSolutions™ General Cytology Preservative.

Each woman provided two samples: one was sent to the Virology Unit at the Medical School of Athens for HPV DNA genotyping (targeting 41 HPV genotypes), while the second was sent to a cytology laboratory for Pap testing, using either conventional cytology or liquid-based cytology (LBC).

Women who tested positive for any high-risk (HR) HPV DNA type and/or had a Pap test result of ≥ASC-US were referred for colposcopy and/or biopsy. For women who tested positive for one or more of the high-risk HPV types 16, 18, 31, 33, or 45, an HPV mRNA test was additionally performed to evaluate oncogenic activity by detecting E6/E7 mRNA transcripts. Since the PreTect HPV-Proofer^®^ assay is specifically designed to detect E6/E7 mRNA from these five HPV types only, mRNA testing was limited to this subset of DNA-positive cases. This stepwise approach was intentional and aimed at optimizing the use of the mRNA assay within its validated detection range. Performing mRNA testing only on DNA-positive samples helped avoid unnecessary testing in women who were unlikely to benefit from it and ensured that all participants included in the mRNA arm of the study had a relevant molecular profile for analysis.

All women were fully informed about the study procedures and provided written informed consent, agreeing to the anonymous use of their data. The study was approved by the Bioethics and Ethics Committee of the Medical School of the National and Kapodistrian University of Athens (NKUA) (Approval No: 1617023003/31 March 2017).

### 2.2. HPV DNA Genotyping

HPV DNA genotyping was conducted using the VisionArray^®^ HPV kit (ZytoVision GmbH, Bremerhaven, Germany), following the manufacturer’s guidelines. This assay enables the qualitative detection and identification of 41 clinically significant HPV genotypes. The method relies on DNA/DNA hybridization, wherein PCR-amplified products bind to specific complementary probes immobilized on a glass microarray slide.

Initially, DNA was extracted using the Maxwell^®^ 16 Viral Total Nucleic Acid Purification Kit (Promega, Madison, WI, USA) on the Maxwell^®^ 16 Instrument (Promega, Madison, WI, USA), which facilitates automated isolation of viral nucleic acids from up to 16 samples with a final elution volume of 50 µL. For sample processing, 1 mL of the preserved cervical specimen was transferred into an Eppendorf tube and centrifuged at 2000 rpm for 12 min. After discarding the supernatant, the resulting pellet was re-suspended in 300 µL and subjected to nucleic acid extraction as per the instrument’s protocol.

PCR amplification was performed using 5 µL of purified DNA combined with 15 µL of HPV PreCise Master Mix—a refined version of the GP5+/GP6+ general primer system.

This reaction amplifies and biotin-labels specific sequences within the L1 region of the HPV genome, with a total reaction volume of 25 µL.

The amplified PCR products were subsequently hybridized onto the VisionArray^®^ HPV Chip 1.0 using the provided hybridization buffer. This microarray contains immobilized DNA probes specific to 41 different HPV genotypes, grouped into three risk categories [16,17]:

- High-risk types: 16, 18, 31, 33, 35, 39, 45, 51, 52, 56, 58, 59
- Probably high-risk types: 26, 34, 53, 66, 67, 68a, 68b, 69, 70, 73, 82IS39, 82MM4
- Low-risk types: 6, 11, 40, 42, 43, 44, 54, 55, 57, 61, 62, 72, 81CP8304, 83MM7, 84MM8, 90, 91

Following hybridization, non-specific binding was eliminated through a series of stringent washing steps. The biotinylated target sequences that remained bound to the chip were then detected using a streptavidin–peroxidase conjugate. Visualization was achieved via tetramethylbenzidine (TMB) staining, which produced blue-colored spots at sites of specific hybridization. The results were analyzed and interpreted using the VisionArray^®^ Analyzer Software 2.0.

### 2.3. HPV mRNA Test

The HPV mRNA test was conducted using the PreTect HPV-Proofer^®^ kit, adhering to the protocol provided by the manufacturer. This assay is intended for the qualitative identification of E6/E7 mRNA transcripts from high-risk HPV types 16, 18, 31, 33, and 45—types that account for the majority of cervical cancer cases globally. The test utilizes real-time NASBA (Nucleic Acid Sequence-Based Amplification), an isothermal RNA amplification technique performed at 41 °C, with detection based on molecular beacon probes. The system employs six molecular beacons, each tagged with one of two fluorescent dyes, to identify E6/E7 mRNA from the specified HPV types and to monitor an internal control (Intrinsic Sample Control—ISC) across three replicate NASBA reactions. A positive test result confirms the presence of E6/E7 mRNA from one or more of the targeted HPV genotypes, whereas a negative result suggests either the absence of detectable transcripts or levels below the assay’s sensitivity threshold.

### 2.4. Cytological Analysis

Cytological evaluation of the samples was conducted using either the traditional Papanicolaou (Pap) smear or liquid-based cytology (LBC). For the conventional Pap smear, a healthcare provider collects cervical cells using a cytobrush and immediately smears them onto a glass slide. This slide is then fixed and forwarded to the laboratory for microscopic assessment to identify abnormal or potentially precancerous cellular alterations. In comparison, the LBC method involves collecting cervical cells into a vial containing a preservative solution, which maintains sample quality and reduces the risk of contamination. The sample is then processed in the laboratory, where the cells are either prepared on a slide or examined directly in their liquid state.

### 2.5. Colposcopy and Biopsy

During a colposcopic examination, the physician employs a colposcope to closely inspect and illuminate the cervix, vagina, and vulva, facilitating the identification of abnormal tissue areas. To improve visualization of suspicious regions, a 3–5% acetic acid solution is applied to the cervix using cotton swabs. Areas that temporarily turn white—a reaction referred to as acetowhitening—are flagged for further evaluation and typically biopsied. In cases where no visible abnormalities are detected, Lugol’s iodine may be used as an additional diagnostic aid. After the colposcopic assessment, a punch biopsy is conducted, during which small tissue samples are extracted from the cervix with biopsy forceps. Multiple samples may be obtained from various sites if deemed necessary. These specimens are then analyzed microscopically by a pathologist to assess for the presence of precancerous changes or cervical malignancy. The physicians performing the colposcopies and biopsies were blinded to the specific HPV genotypes and detailed cytology results but were aware of each patient’s HPV positivity, as per standard clinical referral practice.

### 2.6. Statistical Analysis

All participant data—including demographic information, HPV DNA and mRNA test outcomes, and biopsy results—were compiled and analyzed using SPSS Statistics software Version 27. To explore statistical relationships between categorical variables, the Pearson Chi-square (χ^2^) test and Fisher’s exact test were applied. These tests determine whether there are significant differences between observed frequencies and those expected under the assumption of independence.

## 3. Results

### 3.1. Study Population Characteristics

**Figure 1:**
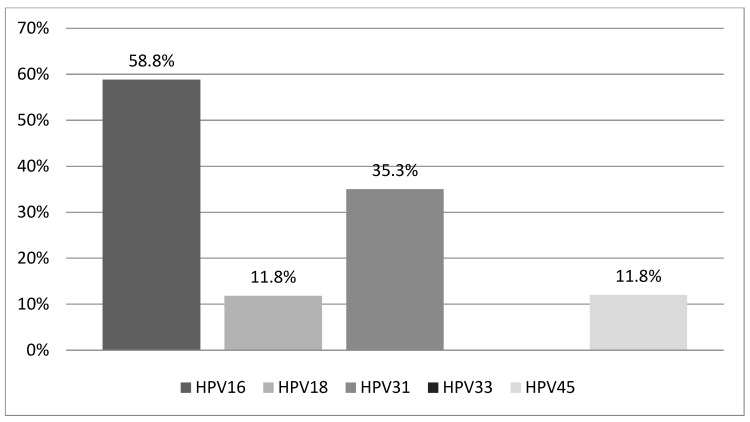
Distribution of HPV types detected among HPV mRNA-positive women (*n* = 17). Abbreviations: HPV, human papilloma virus.

Our study population consisted of 29 Greek women aged 18 to 65 years (mean age: 35.1 ± 10.8 years), all of whom met the previously defined inclusion criteria and tested positive for one or more of the five high-risk HPV types (16, 18, 31, 33, 45) included in the PreTect HPV-Proofer^®^ mRNA assay. Among these women, 17 (58.6%) tested positive for HPV mRNA, while 12 (41.4%) tested negative. Of the 17 HPV mRNA-positive women, 14 (82.3%) had a single HPV infection, and 3 (17.7%) had coinfections involving two HPV types. HPV16 was the most prevalent, detected in 10 women (58.8%), followed by HPV31 in 6 women (35.3%). HPV18 and HPV45 were each identified in 2 women (11.8%), while HPV33 was not detected in any participant. The distribution of HPV types among mRNA-positive women is illustrated in Figure 1.

**Figure 2:**
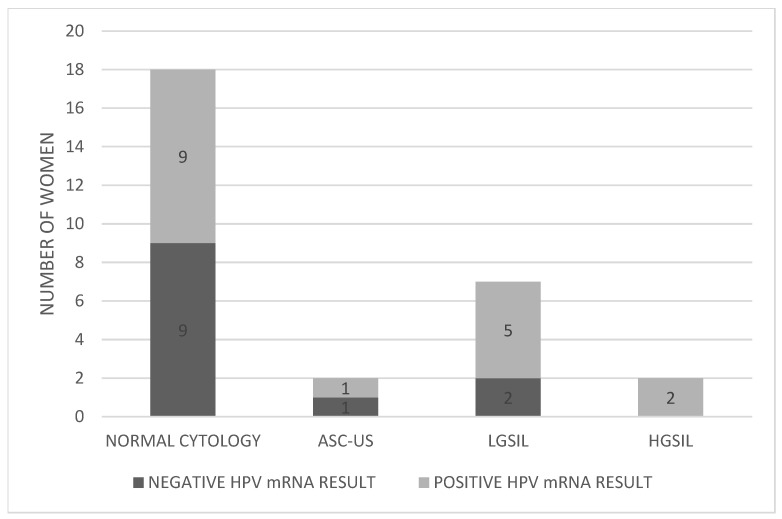
Distribution of HPV mRNA-positive and -negative women according to Pap test results. Abbreviations: ASC-US; atypical squamous cells of undetermined significance, LGSIL; low grade squamous intraepithelial lesion, HGSIL; high grade squamous intraepithelial lesion.

Pap test results for the 29 women revealed that 18 (62.1%) had normal cytology, 2 (6.9%) presented with ASC-US, 7 (24.1%) with LGSIL, and 2 (6.9%) with HGSIL (Figure 2). Table 1 provides detailed results for each individual included in the study.

### 3.2. Comparison of Biopsy Results with HPV DNA Test Outcomes

Histological examination revealed that, among the total 29 HPV DNA-positive women, 11 women (37.9%) had negative biopsy findings, 16 women (55.2%) were diagnosed with CIN1, and 2 women (6.9%) with CIN2. Based on these results, the positive predictive value (PPV) of the HPV DNA test was approximately 55.2% for CIN1 and 6.9% for CIN2, indicating that although the DNA test captured all CIN2 cases, it also included a significant number of false positives, particularly for higher-grade lesions.

### 3.3. Comparison of Biopsy Results with HPV mRNA Test Outcomes

**Figure 3:**
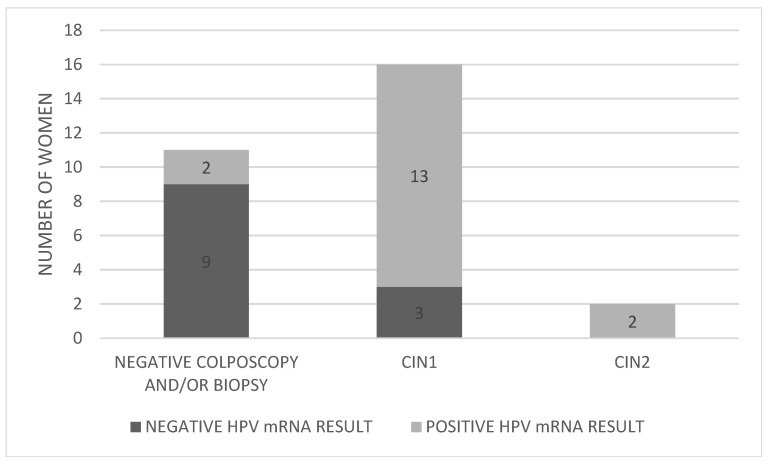
Distribution of HPV mRNA-positive and -negative women by colposcopy/biopsy outcome. Abbreviations: CIN; cervical intraepithelial neoplasia.

The distribution of HPV mRNA-positive and mRNA-negative women by colposcopy/biopsy outcome is presented in Figure 3.

Of the 17 women who tested positive for HPV mRNA, 13 (76.5%) were also found to have CIN1 upon biopsy (cases 3, 5, 7, 8, 10, 15, 16, 19, 20, 21, 22, 23, and 26). Importantly, the two women (100%) diagnosed with CIN2 (cases 2 and 28) were also mRNA-positive, suggesting a strong correlation between mRNA positivity and the presence of higher-grade lesions. This resulted in a PPV of 11.8% for CIN2, while both the clinical sensitivity and negative predictive value (NPV) for CIN2 were 100%. In contrast, among the 12 women who tested negative for HPV mRNA, 3 (25%) were found to have CIN1 (cases 4, 17, and 27).

Therefore, the PPV of the mRNA test for CIN1 was 76.5%, the NPV was 75.0%, and the clinical sensitivity for CIN1 was approximately 81.3%. The Pearson Chi-square test result was χ^2^(2) = 12,205 *p* = 0.002 which indicates statistically significant correlation between the biopsy results of the 29 women and their HPV mRNA test outcomes. Because this test requires that expected frequency in each cell should be ≥5 and in our study, some expected frequencies in the contingency table were below 5, this fact limits the reliability of the Chi-square test. Therefore, Pairwise Fisher’s exact tests (CIN1 vs. negatives, CIN2 vs. negatives, and CIN1 vs. CIN2) were used for more robust analysis in our small sample. In Table 2 are the results of the Pairwise Fisher’s exact tests.

### 3.4. Distribution of HPV Genotypes Among Women with Histological Lesions

HPV16 was the most frequently detected genotype, found in 10 women. Among these, seven had a single infection with HPV16, two had coinfections with HPV18, and one had a coinfection with HPV45. Within this group, two women were diagnosed with CIN2 (one with a single HPV16 infection and one with an HPV16/45 coinfection), four women had CIN1, three had normal biopsy results, and one woman had a negative biopsy.

HPV18 was detected in two women, both in coinfection with HPV16. One of these women had CIN1, while the other had a negative biopsy. HPV31 was detected in six women; five had CIN1 and one had a negative biopsy. HPV33 was not detected in any of the HPV mRNA-positive women. HPV45 was found in two cases—one in coinfection with HPV16 and associated with a CIN2 diagnosis, and the other in a woman with CIN1.

### 3.5. Comparison Between HPV DNA and HPV mRNA Test Performance

Table 3 presents a direct comparison of the diagnostic performance of HPV DNA and HPV mRNA tests for the detection of CIN1 and CIN2. Overall, the HPV mRNA test demonstrated greater clinical specificity, yielding fewer false positives and resulting in a higher PPV for both CIN1 (76.5%) and CIN2 (11.8%). Both tests demonstrated 100% sensitivity for detecting CIN2, as they correctly identified all high-grade lesions. However, the HPV DNA test produced a large number of false positives, particularly for CIN2, with 27 out of 29 women testing positive despite only two cases of confirmed CIN2, which reduced its PPV for CIN2 to just 6.9%.

Conversely, the mRNA test missed three cases of CIN1, resulting in a slightly lower sensitivity (81.3%) and NPV (75.0%) for low-grade lesions compared to its performance in high-grade lesion detection.

## 4. Discussion

The sequential testing strategy used in this study was based on the validated scope of the PreTect HPV-Proofer^®^ assay, which detects E6/E7 mRNA only from HPV types 16, 18, 31, 33, and 45. Therefore, mRNA testing was intentionally limited to DNA-positive women carrying one or more of these five types. While this approach restricted the evaluation of mRNA test performance to a selected higher-risk subgroup, it was designed to reflect real-world triage practice and to avoid the unnecessary application of mRNA testing in women infected with non-targeted HPV types. As such, the study did not aim to assess the assay’s utility in a general screening population but rather to examine its potential added value as a triage tool among women with specific high-risk infections. This targeted design should be considered when interpreting the applicability of the results.

Although HPV DNA tests demonstrate high clinical sensitivity (~90–95%) [18] by detecting a broad range of HPV infections, they may also identify transient infections that are not clinically significant, resulting in lower clinical specificity (~85–90%). In contrast, HPV mRNA tests show slightly lower clinical sensitivity (~85–90%) [19] but higher clinical specificity (~90–95%), as they target the E6/E7 oncogenes, which are typically active in transforming (persistent) infections [12,20].

In our study, 29 women who tested positive for one or more of the following HPV types—16, 18, 31, 33, and 45—via HPV DNA testing were subsequently tested using the PreTect HPV-Proofer^®^ mRNA kit. Seventeen women (58.6%) tested positive for at least one of these HPV types, while twelve women (41.4%) tested negative. HPV16 was the most prevalent type, consistent with findings from other HPV mRNA studies [21,22], followed by HPV31, HPV18, and HPV45. The high detection rates of HPV16 E6/E7 mRNA were expected, considering its strong oncogenic potential primarily driven by overexpression of the E6/E7 proteins.

All 29 women also underwent colposcopy and biopsy, which revealed 2 cases with CIN2, 16 with CIN1, and 11 with negative results. From a clinical perspective, the distinction between CIN1, CIN2, and CIN3 is critical. CIN1 represents mild cervical dysplasia with low-grade squamous intraepithelial lesions (LGSIL), which are more likely of spontaneous regression and minor cancer risk. CIN2 represents a moderate-grade lesion with uncertain potential for progression, which is often considered a threshold for therapeutic intervention [23], while CIN3 is a high-grade lesion (HGSIL) with a strong likelihood of progression to invasive carcinoma if it remains untreated. Due to the small sample size, specific exclusion criteria, and the routine cytological screening of participants, we identified only two cases of CIN2 and none with CIN3 or higher-grade lesions. The two women with CIN2 (Cases 2 and 28) tested positive for HPV mRNA—one for HPV16 and HPV45, and the other for HPV16 alone. Given that these were the only CIN2 cases in the cohort, the mRNA test demonstrated strong clinical sensitivity and specificity for detecting CIN2 lesions and serves as a benchmark for evaluating cervical cancer screening methods.

As reported in the large study by Schiffman et al. [24], while the five-year risk of CIN3+ progression in women with CIN1 and HR-HPV DNA positivity is relatively low (~2%) [25], histopathological classification is highly dependent on the pathologist’s expertise. As a result, more than 10% of biopsies classified as CIN1 by clinical centre pathologists were reclassified as CIN2+ upon expert review. This highlights the need for close monitoring of CIN1 lesions, as discussed in our previous study [26], and supports the utility of HPV mRNA testing in assessing the risk of progression to malignancy.

Among the 16 women diagnosed with CIN1, 13 tested positive for HPV mRNA, indicating active oncogenic transcription associated with histologically confirmed lesions. This supports the test’s high specificity in identifying clinically relevant infections. However, the mRNA test missed three CIN1 cases. There are several plausible reasons for these false-negative mRNA results. First, CIN1 lesions are frequently associated with transient HPV infections that may not involve active expression of the E6/E7 oncogenes. In such cases, although HPV DNA is present, the virus may not have initiated the oncogenic transformation process, resulting in undetectable or absent E6/E7 mRNA transcripts. Second, the viral genome in these lesions may exist in episomal form rather than integrated into the host genome, a scenario typically associated with lower oncogenic potential and reduced transcriptional activity. Additionally, mRNA levels in early-stage or regressing lesions may fall below the assay’s detection threshold.

The Pearson Chi-square test revealed a statistically significant correlation between the biopsy results of the 29 women and their HPV mRNA test outcomes: χ^2^(2) = 12,205 (*p* ≈ 0.002). Pairwise Fisher’s exact tests (CIN1 vs. negatives, CIN2 vs. negatives, and CIN1 vs. CIN2) revealed a significant association between HPV mRNA positivity and CIN1 diagnosis compared to negative biopsy results (*p* ≈ 0.0057). However, the comparisons involving CIN2 did not reach statistical significance, likely due to small sample sizes. These findings support a possible correlation between mRNA positivity and low-grade cervical lesions, though further studies with larger samples are needed to confirm this trend for high-grade lesions.

From a clinical perspective, the above findings do not support the use of the HPV mRNA assay as a stand-alone primary screening method for cervical cancer prevention. Instead, the test is better suited as a triage tool, potentially replacing the Pap test—particularly in cases where a high-risk HPV DNA test is positive for genotypes other than HPV16 or HPV18—to help reduce unnecessary colposcopies. Among the three CIN1 cases with negative mRNA results, two (Cases 17 and 27) were positive for HPV16. According to the 2019 ASCCP guidelines, women testing positive for HPV16 are referred directly for colposcopy and biopsy without triage. Therefore, in such cases, a negative mRNA result should not override the guideline-based recommendation for immediate colposcopic evaluation.

Focusing on HPV16, the most frequently detected type in our cohort, it is notable that 9 out of 10 women with a positive HPV16 mRNA result had either CIN1 or CIN2 on biopsy. Additionally, among nine women with negative biopsy results and positive DNA test results for HPV16/18, only one tested positive on the mRNA test. This implies that if mRNA testing had been used as a triage tool, eight women could have avoided unnecessary colposcopy and biopsy.

In contrast, among five women with CIN1 and positive HR-HPV DNA results for types other than HPV16/18, all tested positive for mRNA (four for HPV31 and one for HPV45).

Cases 15, 19, 21, and 22 illustrate that the mRNA test often identifies a single transcriptionally active HPV type, even when multiple types are detected via DNA testing. The exact mechanism by which each HPV type contributes to lesion development in co-infections remains unclear [27]. However, studies using mRNA expression analysis suggest that a single high-risk HPV type is usually responsible for lesion progression [28,29]. Therefore, HPV mRNA testing, by identifying active oncogenic transcription (rather than just presence of viral DNA), provides higher diagnostic precision and helps avoid overdiagnosis from transient or inactive infections.

Pap test results among the 29 HR-HPV DNA women showed 18 with normal cytology, 2 with ASC-US, 7 with LGSIL, and 2 with HGSIL. Of the 18 women with normal Pap results, 9 had positive HPV mRNA results—and 6 of those had CIN1 on biopsy—indicating that normal cytology alone may miss early dysplasia. Among the two ASC-US cases, one tested mRNA-positive but had no histological lesion, warranting a shorter follow-up interval. Among seven women with LGSIL, five were mRNA-positive, all of whom had CIN1 on biopsy. Since LGSIL often requires further triage, HPV mRNA testing can clarify oncogenic activity and guide management. Although Pap testing detected both CIN2 (HGSIL) cases, mRNA testing confirmed the presence of oncogenic viral activity, reinforcing its complementary value.

In total, out of the 29 women referred for colposcopy/biopsy due to HR-HPV DNA positivity, 8 women (27.5%) (Cases 1, 6, 9, 11, 12, 14, 18, 25) could have avoided these procedures based on negative mRNA results and corresponding negative biopsy findings. Conversely, mRNA testing failed to detect three CIN1 cases (10.3%) (Cases 4, 17, 27), highlighting its lower sensitivity. In these cases, it is possible that the HPV genome existed in episomal form rather than integrated into the host genome, thus escaping detection via mRNA testing while still exerting oncogenic effects [30,31].

A notable limitation of our study is the small sample size, which was influenced by the strict exclusion criteria and the design of our screening protocol. Although we chose these exclusion criteria in order to minimize confounding factors that could affect HPV persistence, immune response, or cervical pathology so that we could have a more homogeneous and clinically interpretable cohort, we acknowledge that these strict criteria may introduce a selection bias and limit the generalizability of our findings. More specifically, our study could have failed to include groups of women that are at higher or lower risk of disease progression.

The limited number of participants, particularly the very small number of CIN2 cases (*n* = 2), significantly restricts the statistical power of our findings and prevents robust estimation of diagnostic metrics such as PPV and sensitivity, especially for high-grade lesions. Furthermore, the small cohort limits the generalizability of the results to the broader Greek female population undergoing cervical cancer screening. As such, the findings should be interpreted with caution and considered exploratory. Larger population-based studies are needed to validate these preliminary observations and determine the broader clinical applicability of HPV mRNA testing as a triage tool.

Another limitation is the narrow detection spectrum of the mRNA kit, which targets only five high-risk HPV types. As a result, it might miss clinically relevant infections with other high-risk types, such as HPV52 or HPV58, potentially reducing the test’s sensitivity and negative predictive value (NPV). Despite this, we selected the PreTect HPV-Proofer^®^ kit because it was the only available mRNA assay at the time that both detected and genotyped these high-risk HPV types.

Lastly, an additional limitation arises from the lack of certain behavioral and sociodemographic data (educational level, economic status, number of sexual partners, condom use, and vaccination status). These factors are known to influence HPV infections, persistence, and progression risk, and their absence limits our ability to perform multivariate risk analysis and should be taken under consideration in future larger cohort studies.

## 5. Conclusions

Our study confirms the high oncogenic potential of HPV16 and HPV31, while highlighting the potential value of mRNA testing in triaging women with a positive high-risk HPV DNA result by filtering out transient infections. Incorporating mRNA testing into cervical cancer screening protocols may enhance the detection of active HPV-driven oncogenesis, thereby identifying women who would benefit from closer monitoring or early intervention. Moreover, mRNA testing could play a valuable role in clinical studies focused on the development of therapeutic HPV vaccines, by determining HPV status before and after vaccination. Further research involving larger cohorts and the use of updated mRNA diagnostic kits—now capable of detecting and genotyping up to 14 high-risk HPV types—will help clarify whether HPV mRNA testing can serve as a complementary, triage, or even stand-alone method for cervical cancer screening.

## Figures and Tables

**Table 1:** Summary of results for the 29 women who underwent HPV mRNA testing.

Case Number	HPV DNA Test	HPV mRNA Test	Pap Test	Colposcopy/ Biopsy
1	16 (High Risk)	Negative	Normal	Negative
2	16 (High Risk), 45 (High Risk)	HPV 16, 45	HGSIL	CIN2
3	16 (High Risk)	HPV 16	LGSIL	CIN1
4	45 (High Risk), 56 (High Risk)	Negative	LGSIL	CIN1
5	16 (High Risk)	HPV 16	Normal	CIN1
6	18 (High Risk)	Negative	Normal	Negative
7	6 (Low Risk), 16 (High Risk), 18 (High Risk), 53 (Probably High Risk), 61 (Low Risk), 62 (Low Risk), 67 (Probably High Risk), 68a (Probably High Risk), 90 (Low Risk)	HPV 16, 18	LGSIL	CIN1
8	31 (High Risk)	HPV 31	Normal	CIN1
9	18 (High Risk), 44 (LowRisk)	Negative	Normal	Negative
10	16 (High Risk), 42 (Low Risk), 73 (Probably High Risk)	HPV 16	Normal	CIN1
11	18 (High Risk), 61 (LowRisk)	Negative	Normal	Negative
12	16 (High Risk)	Negative	ASC-US	Negative
13	18 (High Risk)	Negative	Normal	Negative
14	33 (High Risk)	Negative	Normal	Negative
15	51 (High Risk), 56 (High Risk), 45 (High Risk), 35 (High Risk), 73 (Probably High Risk), 82MM4 (Probably High Risk), 54 (Low Risk), 90 (Low Risk)	HPV 45	Normal	CIN1
16	16 (High Risk), 66 (Probably High Risk), 91 (Low Risk)	HPV 16	Normal	CIN1
17	16 (High Risk), 51 (High Risk)	Negative	Normal	CIN1
18	18 (High Risk), 73 (Probably High Risk)	Negative	Normal	Negative
19	31 (High Risk), 58 (High Risk)	HPV 31	Normal	CIN1
20	31 (High Risk), 73 (Probably High Risk), 44 (Low Risk)	HPV 31	Normal	CIN1
21	31 (High Risk), 59 (High Risk)	HPV 31	LGSIL	CIN1
22	16 (High Risk), 59 (High Risk), 34 (Probably High Risk), 70 (Probably High Risk), 62 (Low Risk), 44 (Low Risk)	HPV 16	LGSIL	CIN1
23	31 (High Risk)	HPV 31	Normal	CIN1
24	31 (High Risk)	HPV 31	ASC-US	Negative
25	18 (High Risk), 66 (Probably High Risk), 62 (Low Risk)	Negative	Normal	Negative
26	16 (High Risk), 66 (Probably High Risk)	HPV 16	LGSIL	CIN1
27	16 (High Risk), 52 (High Risk)	Negative	LGSIL	CIN1
28	16 (High Risk)	HPV 16	HGSIL	CIN2
29	16 (High Risk), 18 (High Risk), 66 (Probably High Risk), 54 (Low Risk), 61 (Low Risk)	HPV 16, 18	Normal	Negative

Abbreviations: CIN; cervical intraepithelial neoplasia.

**Table 2:** Pairwise Fisher’s exact test.

Comparison	Fisher’s *p*-Value	Interpretation
CIN1 vs. Negative	*p* ≈ 0.0057	Statistically significant
CIN2 vs. Negative	*p* ≈ 0.099	Not significant (small numbers)
CIN2 vs. CIN1	*p* ≈ 1.0	Not significant

**Table 3:** Diagnostic metrics comparison.

Metric	CIN1		CIN2	
	HPV DNA Test	HPV mRNA Test	HPV DNA Test	HPV mRNA Test
True Positives	16	13	2	2
False Positives	13	4	27	15
False Negatives	0	3	0	0
True Negatives	0	9	0	12
PPV	55.2%	76.5%	6.9%	11.8%
NPV	—	75.0%	—	100%
Test Sensitivity	100%	81.3%	100%	100%

Abbreviations: PPV, positive predictive value; NPV, negative predictive value; HPV, human papilloma virus.

## Data Availability

The original contributions presented in the study are included in the article, further inquiries can be directed to the corresponding author.
